# Testosterone Promotes the Proliferation of Chicken Embryonic Myoblasts Via Androgen Receptor Mediated PI3K/Akt Signaling Pathway

**DOI:** 10.3390/ijms21031152

**Published:** 2020-02-09

**Authors:** Dongfeng Li, Qin Wang, Kai Shi, Yinglin Lu, Debing Yu, Xiaoli Shi, Wenxing Du, Minli Yu

**Affiliations:** 1 Department of Animal Genetics, Breeding and Reproduction, College of Animal Science and Technology, Nanjing Agricultural University, Nanjing 210095, China; lidongfeng@njau.edu.cn (D.L.); 2016105004@njau.edu.cn (Q.W.); 2017105005@njau.edu.cn (K.S.); luyinglin@njau.edu.cn (Y.L.); yudebing@njau.edu.cn (D.Y.); duwx@njau.edu.cn (W.D.); 2 National Experimental Teaching Demonstration Center for Animal Science, Nanjing Agricultural University, Nanjing 210095, China; shixiaoli@njau.edu.cn

**Keywords:** chicken, myoblasts, PI3K/Akt pathway, proliferation, testosterone

## Abstract

Testosterone (T) is essential for muscle fiber formation and growth. However, the specific mechanism by which T regulates skeletal muscle development in chicken embryos remains unclear. In this study, the role of T in myoblast proliferation both in vivo and in vitro was investigated. Results showed that the T administration significantly increased the ratio of breast muscle and leg muscle. T induced a significant increase in the cross-sectional area (CSA) and density of myofiber and the ratio of PAX7-positive cells in the skeletal muscle. Exogenous T also induced the upregulation of myogenic regulatory factors (MRFs) and cyclin-dependent kinases (*CDK2*)*/Cyclin D1 (CCND1)* and protein levels of androgen receptor (AR), p-Akt and PAX7. Furthermore, T treatment significantly promoted myoblasts cultured in vitro entering a new cell cycle and increased PAX7-positive cells. The mRNA and protein expression of AR and PAX7 were upregulated when treated with T compared to that of the control. The addition of T induced proliferation accompanied by increasing AR level as well as PI3K (Phosphoinositide 3-kinase)/Akt activation. However, T-induced proliferation was attenuated by AR, PI3K, and Akt-specific inhibitors. These data indicated that the pro-proliferative effect of T was regulated though AR in response to the activation of PI3K/Akt signalling pathway.

## 1. Introduction

Myoblast proliferation is an important process during myogenesis and is crucial to skeletal muscle development and regeneration. Sex has critical impacts on skeletal muscle morphology and metabolism in mammals. Hormonal effects of androgens in males or estrogen in females contribute to different skeletal muscle phenotypes and compositions [1]. As androgen shows an anabolic effect on skeletal muscles, T (Testosterone) plays a key role in regulating several biological processes that are resulting essential for the skeletal muscle formation. Previous study showed that exogenous exposure of chicken embryos to T during incubation had influences on a series of physiological and behavioral offspring traits, including growth [2] and immune functions [3]. Muscle satellite cells with T administration caused a remarkable increase in myoblasts number, and formed larger myofibers with more myonuclei [4]. However, the effect of T on chicken myogenesis and the mechanism by which T regulates skeletal muscle development remain unclear.

The formation of the skeletal muscle mass in embryonic stage is crucial for the potential development of skeletal muscle in adult. A series of myogenesis events take place as a result of the activation of specific signalling pathways and up-regulation of MRFs [5]. Studies have reported that T replacement significantly enhanced muscle strength and increased muscle mass in clinics [6,7]. Skeletal muscle regeneration with T was also observed in both young and aged mice post cardiotoxin injury, which resulted in an increase of satellite cells activation and number [8]. Moreover, administration of T filled implants to mice lead to notable increase in weight and fiber CSA of gastrocnemius muscles [9]. Collectively, these studies highlighted the potential role of T administration in restoring aged and atrophying muscle. However, few studies have investigated the regulation of T in myogenesis in embryonic stages, including myoblasts specialization, proliferation, differentiation, hypertrophy and skeletal muscle formation and growth [10]. To clarify the regulatory role of T in myoblast expansion and the underlying molecular mechanism, effects of T on myoblast proliferation in chicken embryos was investigated in the present study.

The mechanism of T’s action, independent of age, was studied using myoblast cells cultured in vitro [11,12]. These studies showed that T accelerated myoblasts differentiation and myotube hypertrophy in myoblast cell cultures. To elucidate the mechanisms of T-induced myogenisis, both cell lines and primary culture cells were co-treated with T and inhibitors for the AR, Akt and mTOR [13,14]. Currently, insulin-like growth factor-1 (IGF-I) and Akt/mTOR pathways are currently accepted as important in regulating T-induced muscle growth and hypertrophy [15,16]. Additionally, Phosphoinositide 3-kinase (PI3K) inhibitor (LY294002) was recently shown to attenuate T-induced myoblast differentiation and myotube hypertrophy, demonstrating the importance of downstream signalling in mediating the function of T [14].

Myoblast development regulated by androgens such as T and 5-α-dihydrotestosterone (DHT) was mediated via several myogenic signalling pathways. Effects of the T, an agonist of the AR, on skeletal muscle development are thought to be mediated via AR. In addition, a number of evidences indicated that T influences myogenesis through either AR-dependent or AR-independent pathways. It has been reported that PI3K/Akt activation is essential in regulating myoblast proliferation and hypertrophy [13,17]. The pro-proliferation of C2C12 myoblasts was achieved by activation of PI3K/Akt signalling pathway. Furthermore, the proliferation of C2C12 myoblasts and chicken myoblasts were inhibited by LY294002 (PI3K inhibitor) [9,18].

However, T’s effects on myoblast proliferation and skeletal muscle growth during embryonic development in chickens are needed to be determined. Some studies suggested that the induction of myogenic factors in response to T stimulation is responsible for these interactions. However, the exact mechanism by which T regulates myogenic factors expression during skeletal muscle development is not clarified. Specifically, the downstream pathways of T that leads to the expression of myogenic factors is unclear. Therefore, the purpose of this study was to investigate the molecular mechanisms of T-induced myoblast proliferation in chicken embryos.

## 2. Results

### 2.1. Exogenous T Increased the Skeletal Muscle Mass in Chicken Embryos

**Figure 1:**
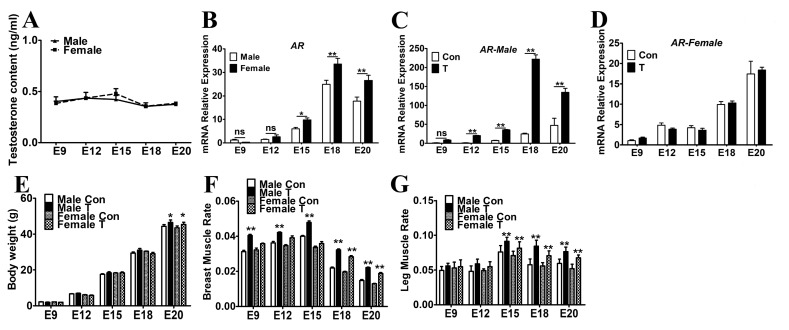
The measurement of T level, muscle mass and *AR* expression. (**A**): The content of endogenous T in embryonic chickens. (**B**): *AR* mRNA expression in embryonic chickens. *AR* mRNA expression in male (**C**) and female (**D**) chicken embryos after injection of T. The body weight (**E**), the ratio of breast muscles (**F**) and leg muscles (**G**) in embryonic chickens with or without T treatment. Data are presented as the means + SE. Asterisk (*) represents statistically different (*p* < 0.05). *p* < 0.01 is shown as **. *n* = 20.

The concentration of endogenous T was measured in chicken embryos by radioimmunoassay and there was no significant differences of T contents in male and female skeletal muscles during the whole stage of embryonic development (Figure 1A). As an androgen receptor, AR can directly respond to changes in hormone levels. The mRNA expression of *AR* in female chickens was obviously higher than that of males at embryonic day 15 (E15) to E20 (*p* < 0.05, Figure 1B). Then, T (10 ng/egg) was injected to fertilized eggs from embryonic day 0 (E0) and the skeletal muscles in different embryonic stages were collected for further ananlyses. Following T injection, the mRNA expression of *AR* was significantly increased in male chicken embryos from E12 to E20 compared to that of the control group (*p* < 0.01, Figure 1C), while there were no significant changes in female chicken embryos (*p* > 0.05, Figure 1D). Therefore, in the following experiment, muscle tissues of male chicken embryos were used.

The weights of T-injected chicken embryos were significantly increased compared with the controls at E20 (Figure 1E). Although there was no significant difference in the body weight of E9-E18 chicken embryos, the difference in body weight increased following the embryonic development. In E20, accumulative impacts of T on body weight was obtained in both male and female embryos (*p* < 0.05). Moreover, administration of T also influenced the proportion of skeletal muscles in chicken embryos. For instance, exogenous T administration led to significant increases in the ratios of breast muscles at E9-E20 of the male embryos and E18-E20 of the female embryos (Figure 1F); the ratios of leg muscles at E15-E20 of both male and female embryos (Figure 1G). These results indicated that T influences the muscle mass growth in chicken embryos.

### 2.2. Exogenous T Augmented the Skeletal Muscle Fiber Proliferation

**Figure 2:**
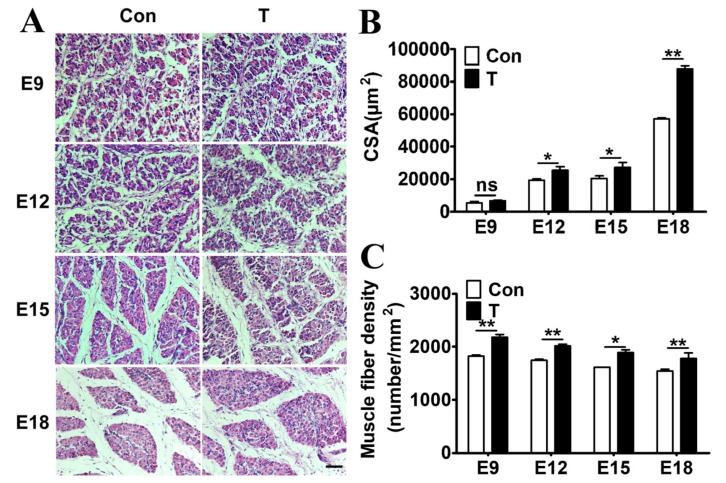
Effect of T treatment on myofiber properties. (**A**): HE staining of paraffin sections of leg muscles at E9–E18. The cross-sectional area of myofibers (**B**) and myofiber density (**C**) was measured. Scale bar: 100μm. Data are presented as the means + SE. Asterisk (*) represents statistically different (*p* < 0.05). *p* < 0.01 is shown as **.

Development of myofibers in chicken embryos was observed using HE (hematoxylin and eosin) staining. Results showed that the CSA and the density of myofibers in T-injected chicken embryos were significantly higher than those in control group (Figure 2A). A significant increase in muscle fiber fusion was also observed in each period, indicating that T treatment promoted myoblast proliferation and myofiber fusion. The CSA of the muscle fibers in the T-treated group increased significantly at E12- E18 (*p* <0.05, Figure 2B). Muscle fiber density of the T-treated group was higher than that of the control group at each stage (*p* <0.05, Figure 2C). HE staining demonstrated that the administration of T led to a significant increase in the number and the diameter of myofibres in skeletal muscles compared with the controls. As a result, growth of skeletal muscle was achieved by increasing the number and the size of myofibers induced by T.

### 2.3. Exogenous T Upregulated the Expression of MRFs and Cell Cycle Related Genes

**Figure 3:**
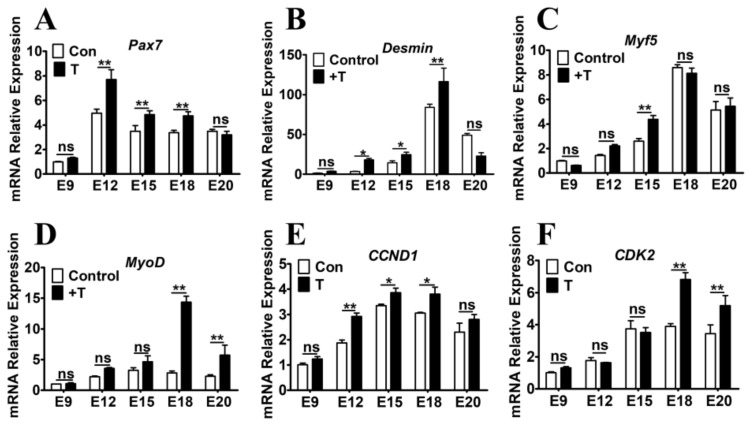
Effect of T treatment on genes expression in embryonic chicken skeletal muscles. (**A–F**): The mRNA expression of *Pax7*, *Desmin*, *Myf5*, *MyoD*, *CCND1* and *CDK2* was detected in the skeletal muscles from the T-treated and control chickens. Data are presented as the means + SE. Asterisk (*) represents statistically different (*p* < 0.05). *p* < 0.01 is shown as **.

To detect the expression of MRFs during myogenesis, the genes expression of embryonic chicken muscles was measured by quantitative real-time PCR (qRT-PCR). mRNA expression of the MRFs was upregulated following T treatment compared that of the controls (Figure 3). Specifically, administration of T resulted in significant upregulation of *Pax7*, *Myf5*, *Desmin* and *MyoD* expressions, indicating that the expression of these genes was regulated by T during myogenesis. In addition, the expression level of *CCND1*/*CDK2* was higher in the T administered group than that of the control group (Figure 3).

### 2.4. Exogenous T Promoted the Protein Expressions of AR, p-Akt and PAX7

**Figure 4:**
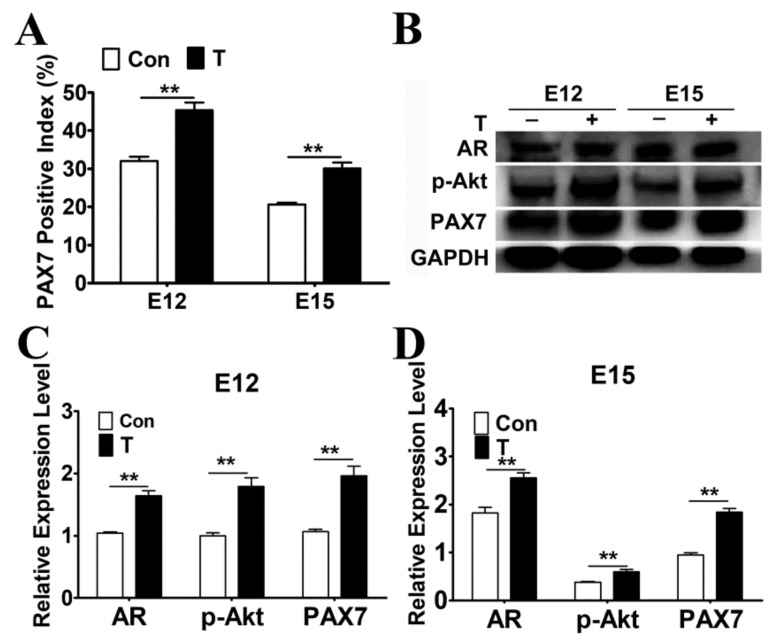
Effect of T treatment on the expression of PAX7 in skeletal muscle. (**A**) The positive percentage of PAX7 positive myoblasts in skeletal muscles. (**B–D**) Protein expression of AR, p-Akt and PAX7 after T treatment was measured by western blotting. Asterisk (*) represents statistically different (*p* < 0.05), ** indicates that the difference is extremely significant (*p* < 0.01).

In order to study the effect of T on myoblast proliferation, the leg muscles of E12 and E15 were subjected to PAX7 immunofluorescence staining. As shown in Figure 4A, the positive percentage of PAX7 in T treatment group in both E12 and E15 was significantly increased compared with that of the control (*p* < 0.01), indicating that T actually promoted the proliferation of chicken embryonic myoblasts. To verify whether the role of PI3K/Akt in myoblast proliferation was associated to AR, AR protein levels were measured by western blotting. The result showed that the exogenous T significantly increased the protein expression of AR, p-Akt, and PAX7 in skeletal muscles of E12–E15 chicken embryos (*p* < 0.01, Figure 4B–D).

### 2.5. Effects of T and Inhibitors on the Cell Cycle of Myoblasts

**Figure 5:**
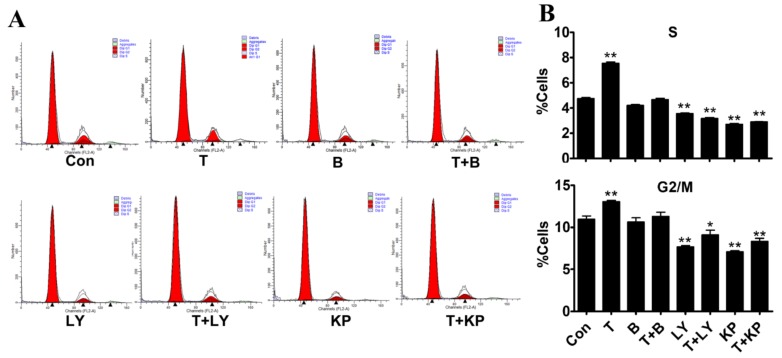
Effect of T and inhibitors treatment on cell cycle of myoblasts cultured in vitro. (**A–B**): Cell cycle of myoblasts treated with T and/or inhibitors (Bicalutamide, B; LY294002, LY; KP372-1, KP) was detected by flow cytometry. The percentage of S or G2/M stage cells was measured in different treatment compared with control (Con). A minimum of 15,000 myoblast cells were analysed for each sample. Values are means + SE of four independent experiments with triplicate determinations. Asterisk (*) represents statistically different (*p* < 0.05), ** indicates that the difference is extremely significant (*p* < 0.01).

To measure the contribution of PI3K/Akt in T-induced myoblast proliferation, specific inhibitors of AR, PI3K, and Akt were used before T treatment, to inhibit the activities of AR, PI3K and p-Akt, respectively. Then, the cell cycles of myoblasts in each group were assessed by flow cytometry. The results showed that T treatment significantly increased S and G2 phase cell populations by 5.05% and 2.95% respectively, when compared to the control group (Figure 5). In contrast, the percentage of S and G2 phase was significantly reduced in myoblasts co-treated with T and LY294002 or KP372-1 (Figure 5). The activation of the PI3K/Akt signalling by T resulted in accumulation of myoblasts in the S and G2 phases, leading to a rapid proliferation. However, the mitosis inducing effect of T in myoblasts was blocked by inhibitors, which suppressed myoblast proliferation.

### 2.6. Effects of T and Inhibitors on the Expression of PAX7 in Myoblasts

**Figure 6:**
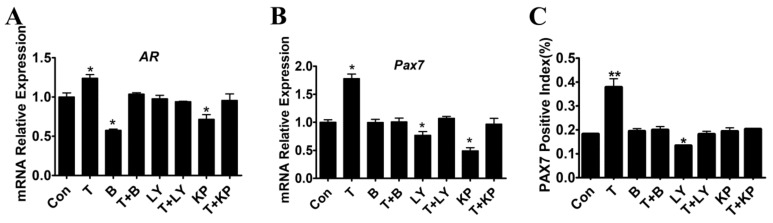
Effect of T and inhibitors on the expression of PAX7 in cultured myoblasts. (**A–B**): The mRNA expression of *AR* and *Pax7* after T and/or inhibitors treatment was measured by qRT-PCR. (**C**) The positive index of PAX7 in cultured myoblasts treated with T and/or inhibitors. Asterisk (*) represents statistically different (*p* < 0.05), ** indicates that the difference is extremely significant (*p* < 0.01).

Furthermore, a similar pattern of T-induced PAX7 expression was observed in vitro myoblast culture model. Myoblasts were treated with T and/or Bicalutamide (50 μM), LY294002 (10 μM) or K372-1 (1 μM) for 24 h before the measurement. T-induced up-regulation of *AR* and *Pax7* mRNA was significantly reduced by inhibitors compared with control (*p* < 0.01), supporting the AR-dependent activation of T-mediated myoblast proliferation (Figure 6A,B). Then the protein expression of PAX7 in myoblasts treated with T and/or inhibitors was measured by immunofluorescence staining. The ratio of PAX7 positive cells in T-treated group was significantly higher than that of control group (*p* < 0.05, Figure 6C). As shown in Figure 6, the increased positive index of PAX7 in myoblasts induced by T was significantly suppressed when co-treated with AR, PI3K or Akt inhibitor (*p* > 0.05). The above results further indicated that T is able to stimulate myoblast proliferation in vitro, while inhibitors blockaded the pro-proliferative effect of T on myoblasts.

### 2.7. Effects of T on the Activation of PI3K/Akt Signalling Pathway

**Figure 7:**
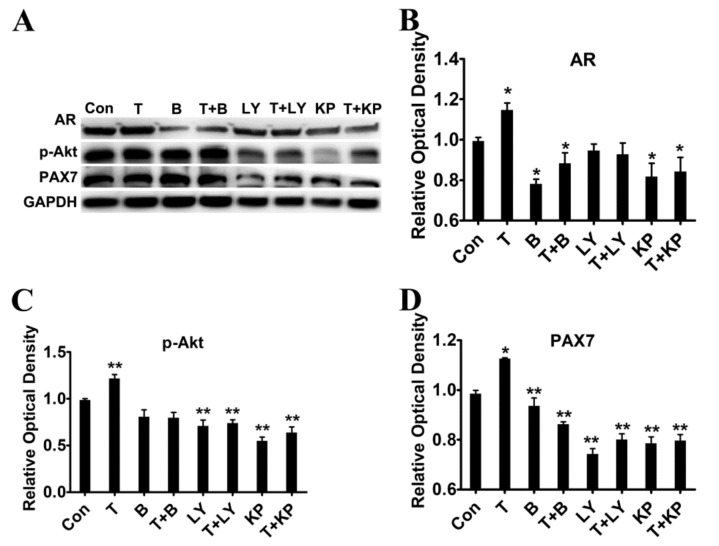
Effect of T and inhibitors on protein expression of AR and PAX7. (**A–D**): Protein expression level of AR, p-Akt and PAX7 after T and/or inhibitors treatment. Asterisk (*) represents statistically different (*p* < 0.05), ** indicates that the difference is extremely significant (*p* < 0.01).

To evaluate whether the T-induced proliferation is responsible for the activation of PI3K/Akt signalling pathway, myoblasts were treated with inhibitors in the presence of T for 24 h. Bicalutamide (AR antagonist) significantly reduced the protein expression of AR and eliminated the upregulated effect of T compared with control (*p* < 0.01, Figure 7A,B). Phosphorylated Akt protein level in myoblasts was increased by addition of T, while it’s decreased by co-treatment with inhibitors compared with control (*p* < 0.01, Figure 7C). The increase in PAX7 protein level induced by T treatment was abolished when cells were co-treated with inhibitors (Figure 7D). Co-treatment with inhibitors suppressed the upregulation of PAX7, suggesting that T-induced proliferation of myoblasts in chickens was mediated by AR and PI3K/Akt signalling pathway.

## 3. Discussion

Skeletal muscle is the main source for proteins in animals and is one of the main organs involved in energy acquisition and maintenance. The weight and size of muscle mass respond very rapidly to hormone stimulation during anabolism or catabolism [19,20]. Previous study showed that androgens promoted muscle growth by maintaining myoblasts in the proliferate state and delaying the differentiation process [8]. The impact of T on muscle growth is thought to be mediated though AR. However, cellular and molecular mechanisms responsible for the action of T on myogenesis in embryonic chickens remain unclear.

Reports indicated that T can increase muscle mass and decrease the amount of fat in a dose-dependent manner in young and older men [6,9,21]. The increase in muscle mass was due to hypertrophy of myofibers with response to T [13]. However, since the growth of chicken embryos are outside the hen’s body when hatching, the development of chicken embryos was affected easily and greatly by exogenous hormones. In addition, androgen has vital impacts on muscle mass and myofiber phenotypes in chickens, which will affect the production and meat quality in adult chickens. Therefore, further studies are needed to determine the molecular mechanisms of anabolic androgen action in muscle development in embryonic chickens. To analyze the role of T in embryonic muscle development, the exogenous T was injected into chicken embryos, then the proliferation of myoblasts and skeletal muscle growth was detected. Results indicated that the ratio of breast muscles and leg muscles significantly increased after T injection (*p* < 0.01). Under T treatment, the CSA and density of myofibers was also increased significantly (*p* < 0.05), indicating that T enhanced myoblast proliferation and promoted skeletal muscle formation. Increased muscle mass, CSA and myoiber density, combined with increased ratio of PAX7 positive myoblasts were achieved by addition of T. Taken together, these results confirmed that exogenous T can effectively promote skeletal muscle growth by inducing myoblast proliferation in chicken embryos.

T and its synthetic homologues have been used clinically and illegally to increase muscle mass [6,22]. In response to T administration, the number of proliferating cell nuclear antigen (PCNA) positive satellite cells increased in human skeletal muscle [22]. Satellite cells expressing the PCNA indicated that T can promote the entry of satellite cells into the cell cycle [22]. T might also favor the commitment of pluripotent precursor cells into myotubes and inhibit adipogenic differentiation [4]. T was also able to stimulate the mitotic activities of myoblast cells in culture systems [23,24]. The present study supported a crucial role of T in enhancing myogenic proliferation with in vitro myoblast cell culture model. Different mechanisms are involved in T-induced skeletal muscle hypertrophy, including promotion of myoblast proliferation, satellite cells entry into cell cycles, commitment of pluripotent mesenchymal cells to myogenic lineage, and activation of intracellular AR [13,14,23]. In males, androgens acted through AR to regulate multiple signalling pathways that control muscle mass, strength and fatigue resistance [1]. Studies of muscle-specific ARKO mouse models provided evidence that a direct role of AR in hind limb muscles was to regulate their strength and fiber type distributions, and it was also a major muscle quality determinant [11,19]. In this study, the expression of endogenous *AR* mRNA increased gradually following embryonic development, and it was higher in females than that of males (*p* < 0.05). *AR* mRNA and protein expression levels increased significantly in male skeletal muscles after T treatment (*p* < 0.05), indicating that T promoted myoblast proliferation and myofiber development by regulating the expression of AR.

In addition to regulating gene expression of *AR*, T also produced a rapid, non-transcriptional response involving membrane-linked signal transduction pathways, PI3K/Akt cascade signal was shown to be crucial for satellite cells and myoblasts to transition from G1 to S progression [18]. Using the in vitro model, the signalling pathways that AR may regulate were explored. The PI3K inhibitor LY294002 and the Akt inhibitor KP372-1 were used to detect the positive index of PAX7 cells, which also inhibited the proliferative effect of T. The inhibitors effectively inhibited protein expression of PAX7 and arrested muscle growth with T. Importantly, T-induced myoblasts proliferation was mediated via PI3K/Akt pathway. Inhibition of this pathway abrogated the potent influence of T. This study supported the previous works that PI3K/Akt pathway was implicated in T’s action in L6 myoblasts [8,17] and in human skeletal myoblasts [15,25]. Another study reported that the Akt signalling downstream of PI3K was restored by T administration in aged mice [9]. The present study further supported that the action of T in myoblasts proliferation was mediated via PI3K/Akt pathway, especially where reduction in Akt activation were previously reported in C2C12 culture model [26]. The main focus of this study was to investigate the cellular and structural changes observed in chicken skeletal muscle formation during embryogenesis in response to T administration. Further studies are needed to determine the cellular and molecular pathways by which T exerts its action on skeletal muscle development after hatching.

In conclusion, these results demonstrated here that T stimulated myoblast proliferation and promoted dramatic skeletal muscles growth in chicken embryos. Myoblasts increased in number and size and myogenic factor gene expression was upregulated following T administration, suggesting that T participated in early embryonic skeletal muscle development. Collectively, this study implicated pivotal roles of T in regulating myogenesis and stimulating skeletal muscle growth in embryonic chickens through AR- mediated PI3K/Akt signalling pathway.

## 4. Materials and Methods

### 4.1. Experimental Animals and Tissue Samples

Experiments were approved by the Animal Care and Use Committee of Nanjing Agricultural University. Incubation of fertilized chicken eggs (*Arbor Acres Broilers*) was conducted in an incubator (GRUMBACH BSS160, Mücke, Germany) at condition of 38.5 °C and 60% humidity. Injections were made directly into the albumen of the fertilized eggs with 100 ng/mL T in 100 μL per embryo from embryonic day 0 (E0) of incubation and once every three days. The control eggs were injected with 100 μL PBS only. Then, the injection ports were sealed with a melted wax block for further incubation to relative stages. The samples of skeletal muscles were carefully dissected from E9–E20 chicken embryos for further analysis.

### 4.2. T Radioimmunoassay

Female and male leg muscle tissues were taken as experimental samples in five periods. Each milligram of muscle sample was mixed with 200 μL of PBS homogenate, centrifuged at 3500 rpm for 25 min, and the supernatant was stored at −20 °C. Round-bottom polypropylene tubes were numbered and added with samples according to the order of the kit. All reagents and samples were mixed and equilibrated at room temperature (RT). The mixture was allowed to stand at RT for 15 min, centrifuged at 3500 rpm for 15 min, the supernatant was aspirated, and the radioactivity count of each sedimentation tube was measured on a gamma counter.

### 4.3. Hematoxylin-Eosin (HE) Staining

The muscle samples were carefully collected from E9–E20 chicken embryos and fixed in 4% neutral paraformaldehyde overnight. Tissues were rinsed in PBS for 3 h, and then dehydrated in a graded series of ethyl alcohol and embedded in paraffin wax. The transverse serial sections of 5 μm thickness were cut and stained with hematoxylin and eosin for morphological examination. Neutral gum was added and covers section with a cover slip. The morphology of skeletal muscles was observed and muscle fiber CSA (μm^2^) was determined under Eclipse 80i microscope (Nikon, Tokyo, Japan) and the pictures were captured with a digital camera (DS-Fi1, Nikon, Tokyo, Japan). Five fields in each section were randomly selected, and myofiber density was evaluated based on the number of myofiber per mm^2^. Three samples were taken from each group in each period, and five sections were selected for each sample to be photographed and counted to obtain data.

### 4.4. Cell Culture and Treatments

Myoblasts were isolated and purified using a differential attachment technique as previously described [18]. In brief, the leg muscles from E9 chickens were carefully dissected. Then samples were cut into 1 mm^3^ small tissue pieces and digested into single cell suspension with 0.25% trypsin in DMEM. Myoblasts were then expanded in DMEM medium supplemented with 15% FBS and 1% penicillin-streptomycin in an incubator with 5% CO_2_ at 37 °C. The culture medium was changed every other day, and cells were passed when approximately 80% confluence was attained. The second passage myoblasts were treated with 1 μM T (Tocris Bioscience, Bristol, UK) with or without various inhibitors. Specific inhibitors including 50 μM AR inhibitor (Bicalutamide, Shanghai Civi Chemical Technology, Shanghai, China), 10 μM PI3K inhibitor (LY294002, Sigma Aldrich, St. Louis, MO, USA), and 1 μM Akt inhibitor (KP372-1, Echelon, Logan, UT, USA) were added into culture medium for 30 min before T addition. DMSO (0.1%) was used as the vehicle control. All treatments were conducted at 0 h and maintained in culture medium for 24 h.

### 4.5. RNA Isolation and Qualitative RT-PCR

Myoblasts were collected from three wells at each group and the experiments were repeated three times for each sample. Total RNAs from skeletal muscles and cultured cells were isolated with Trizol reagent (Invitrogen, Carlsbad, CA, USA) by following the manufacturer’s instructions. Quality and purity were determined by the Nanodrop-2000 spectrophotometer (Thermo Scientific, Wilmington, DE, USA). 1 μg of each RNA sample was reverse transcribed to cDNA after DNase treatment by using 5× All-In-One RT MasterMix (Applied Biological Materials Inc. Richmond, BC, Canada). Real-time quantification of RNA targets was performed using SYBR Premix Ex Taq kit (Takara, Dalian, China) on an ABI7500 fast thermal cycler (Applied Bio-systems Biosystems, CA, USA). The 20 μL reaction mixture contained: 2 μL cDNA, 10 μL 2× SYBR Green SuperReal PreMix (ABM Inc. Richmond, BC, Canada), 0.5 μL of each primer, and 7 μL RNase-free H_2_O. PCR amplification was performed as follows: 94 °C for 30 s, followed by 39 cycles at 94 °C for 15 s, 58–62 °C for 30 s. Expression level was normalized to *β-actin* expression. Results were analyzed using the ^ΔΔ^*C*t method. Each RNA sample was analyzed in triplicate determinations. Primer sequences are listed in Table 1.

### 4.6. Immunofluorescence Staining

Myoblasts were plated on 35 mm cover slips and treated with 1 μM T for 48 h in serum free culture medium, and then were fixed in 4% paraformaldehyde for 10 min at RT. Myoblasts were permeabilized with 0.1% Triton X-100 and rinsed with PBS, blocked with PBS-1% BSA for 1 h, and incubated with PAX7 (1:200, DSHB, Iowa City, IA, USA) at 4 °C overnight. After incubated with FITC goat anti-mouse IgG (1: 1000) secondary antibody (KPL Inc., Gaithersburg, MD, USA), the nuclei were counterstained with DAPI. Cells were analyzed immediately after staining using a confocal laser microscope (Zeiss LSM 700; Carl Zeiss AG, Germany).

### 4.7. Flow Cytometry Analysis (FACS)

FACS was performed as previously described [18]. Briefly, myoblasts were collected and fixed with 70% ethanol at −20 °C for 30 min after washing with ice-cold PBS. Then, myoblasts were incubated with staining solution (50 mg/mL propidium iodide, 0.1% Triton X-100 and 100 mg/mL RNase A in PBS) for 15 min in dark. The stained myoblasts were analysed by flow cytometry (BD FACS CELLULAR). A minimum of 15,000 cells were analysed for each sample. The assay was repeated three times.

### 4.8. Western Blotting Analysis

Myoblasts were harvested and lysed in RIPA buffer supplemented with protease inhibitors (Complete, Roche) and 0.2 nM PMSF. Proteins (20 μg) were denatured and subjected to 10% SDS/PAGE electrophoresis. After transfer to PVDF membrane, blots were treated according to standard procedures. Probing of the blots was performed using phosphor-Akt (1:1000, Cell Signaling Technology, Beverly, MA, USA), anti-androgen receptor (AR, 1:1000, DSHB, Iowa City, IA, USA), PAX7 (1:1000, DSHB, Iowa City, IA, USA) and GAPDH antibody (1:5000, Novus Biologicals, Littleton, CO, USA) as primary antibodies. After 1 h incubation with anti-mouse or anti-rabbit peroxidase-conjugated second antibody (1:5000, Santa Cruz, Dallas, TX, USA), blots were developed by enhanced chemiluminescence (GE Healthcare, Chicago, IL, USA).

### 4.9. Statistical Analysis

The results are expressed as the means ±SE of at least three independent biological replicates. The images show a representative result of experiments that were all repeated at least three times. Data were analyzed by ANOVA and Duncan’s multiple range tests using the SAS 9.0 software. *p* < 0.05 was considered as significantly different.

## Figures and Tables

**Table 1:** Primer sequences for genes of PCR analysis.

Gene	Primer Sequence (5′–3′)	Ref. Sequence Number	Amplicon Length (bp)
*β-actin*	F: ATG AAG CCC AGA GCA AAA GA	NM_205518	223
	R: GGG GTG TTG AAG GTC TCA AA		
*AR*	F: AAG AAG CTG GGC AGT CTG AA	NC_006091.5	214
	R: AGC AGG TTG GAG AAG GAG TC		
*Pax7*	F: AGT TCG ATT AGC CGT GTG CT	NM_205065	185
	R: CTC TTC AAA GGC AGG TCT GG		
*Myf5*	F: CCC ATC CGA GCT CTT CTA TG	NM_001030363	223
	R: CAT GGT GGT GGA TTT CCT CT		
*MyoD*	F: ATG TCC CAT ACT GCC TCC AG	NM_204214	235
	R: GTC TTG GAG CTT GGC TGA AC		
*Desmin*	F: CTG CTT TCA GAG CTG ACG TG	XM_004942838	155
	R: CTG GAT GTG CTG CTC CTG TA		
*CDK6*	F: TTG TTT GAT GTG TGC ACC GT	NM_001007892	165
	R: AGT CCC CGA AAC AGC TGA AG		
*Cyclin D1*	F:GTG CGT GCA GAA GGA AAT CT	NM_205381	225
	R:GCG GTC AGA GGA ATC GTT TC		
